# Time-energy measured data on modern multicore systems running shared-memory applications

**DOI:** 10.1016/j.dib.2019.104670

**Published:** 2019-10-16

**Authors:** Dumitrel Loghin, Yong Meng Teo

**Affiliations:** National University of Singapore, Singapore

**Keywords:** Time-energy performance, Multicore system, Shared-memory, Amdahl's law, Gustafson's law, Analytic model

## Abstract

This article presents execution time and energy data collected from modern multicore systems running shared-memory applications, analyzed using our analytic models. While the full data sets and source code are available on Github, this data-in-brief article includes some samples and describes the experimental setup.

**Table:** Specifications Table

Subject area	*Computer Science*
More specific subject area	*Parallel Systems Performance*
Type of data	*Tables and figures*
How data was acquired	*Power and energy data were collected with a Yokogawa WT210 power meter*
Data format	*Raw and filtered*
Experimental factors	*Execution time and energy data were collected while the hardware system was running only the target shared memory application and the operating systems. The measured data includes noise from the operating system. There is no pretreatment of samples or data.*
Experimental features	*- Power and energy data were collected with a Yokogawa WT210 at a rate of one sample per second* *- Execution time represents wall clock time and is measured in Linux using/usr/bin/time*
Data source location	*Singapore*
Data accessibility	*The data and source code associated with this paper are available on Github:*https://github.com/dloghin/multicores-time-energy

**Table:** 

**Value of the Data** • This set of data includes execution time and energy measurements of up to ten shared-memory applications covering multiple domains on a wide range of modern multicore systems. These systems include both high-performance and low-power, homogeneous and heterogeneous, and are representative for server, desktop and mobile domains. • The data can be used to understand the time and energy performance of modern shared-memory multicore systems. It can serve as a reference for other researchers in the domain. • The source code implements the models described in our work [1,2] and serves as a starting point for researchers, developers and system designers

## 1 Data

In this article we present the time-energy data measured for shared-memory applications running on modern multicore systems [1,2]. We provide two main data sets for each system and application, (i) measured, or raw, time-energy values as shown in Table 3, Table 4, Table 5, Table 6, Table 7 and (ii) model output as shown in Table 8, Table 9. Table 3 presents measured data on homogeneo Table 4, Table 5, Table 6 present measured data on heterogeneous multicores with static OpenMP scheduling when big, little and all cores, respectively, are used. Table 7 presents measured data on heterogeneous multicores with dynamic OpenMP scheduling when all cores are used. Table 8 shows model's output per system and application, while Table 9 presents a summary of model accuracy per system for all applications and speedup laws used, with respect to the sequential fraction and energy savings. The data in Table 8, corresponding to Amdahl's law [3], is plotted in Fig. 2. The corresponding data derived with Gustafson's law [4] is plotted in Fig. 3.

## 2 Experimental design, materials and methods

### 2.1 Setup

**Fig. 1:**
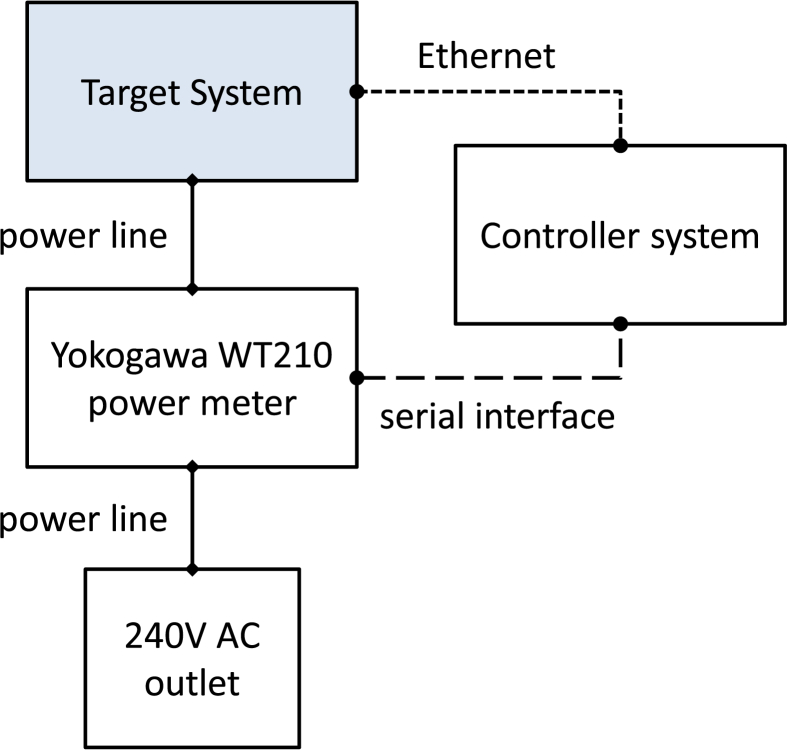
Experimental setup.

The experimental setup is depicted in Fig. 1. To collect power and energy, we use a Yokogawa WT201 power meter connected to the 240V AC power line. A controller system is used to start the experiments and collect execution and energy data from the target system. The power and energy samples are collected once per second. Table 1 summarizes the characteristics of the target systems used in our measurements.

**Table 1:** Systems.

System	CPU	Cores	Frequency [GHz]	Memory [GB]
AMD	AMD Opteron K10	48	2.10	64 (NUMA)
ARM	Cavium ThunderX (64-bit ARM)	48	2.00	128 (UMA)
Xeon	Intel Xeon E5-2630 v4	10 (20 HT)	2.20	64 (UMA)
i7	Intel Core i7-6700	4 (8 HT)	3.40	16
Pi3	ARM Cortex-A53	4	1.20	1
XU3	ARM big.LITTLE HMP (ARM Cortex-A15 + ARM Cortex-A7)	8 (4 + 4)	2.00	2
TX2	HMP (Denver + ARM Cortex-A57)	6 (2 + 4)	2.04	8

Table 2 summarizes the shared-memory applications with their input parameters, as used for collecting the measurements. These applications are selected from well-known benchmarking suites, such as NPB [5], Rodinia [6], Parsec [7] and Mantevo [8]. In addition to the first seven applications presented in our research work [1,2], we provide data for CloverLeaf (CL), miniFE (FE) and miniGhost (GH) benchmarks from Mantevo suite [8], running on Xeon, i7 and Pi3.

**Table 2:** Applications.

Application	Benchmark Suite	Input Size	OpenMP Scheduling
EP (Embarrassingly Parallel)	NPB [5]	Class C (Random-number pairs: 2^32^)	default
BT (Block Tri-diagonal Solver)	NPB [5]	Class C (Grid size: 162 × 162 x 162, Iterations: 200)	static
SP (Scalar Penta-Diagonal solver)	NPB [5]	Class C (Grid size: 162 × 162 x 162, Iterations: 400)	Static
LV (LavaMD)	Rodinia [6]	Boxes1d: 24	default
KM (Kmeans)	Rodinia [6]	n = 1,000,000 m = 34 k = 5	static
PF (Pathfinder)	Rodinia [6]	Width (rows): 900,000, Steps (columns): 500	default
BS (BlackScholes)	Parsec [7]	4,000,000 options	default
CL (CloverLeaf)	Mantevo [8]	Grid size 1000, end_time = 30.0	default
FE (miniFE)	Mantevo [8]	nx = 150	default
GH (miniGhost)	Mantevo [8]	nx = 100, num_tsteps = 1000	default

### 2.2 Measured data

Measured time-energy data consists of seven columns, as shown in Table 3 for EP execution on Xeon. Each row represents the execution on a number of cores of the given application on the given system. The columns represent the number of nodes, number of cores per node, the core clock frequency of the cores, the execution time in seconds (s), the energy in Watts-hour (Wh) and Joules (J), and the average power consumption in Watts (W). The number of nodes is always one because these experiments are run on single-node shared-memory multicore systems. To apply our models [1,2], the key columns to consider are Cores, Time and Energy.

**Table 3:** Raw time-energy measurements (EP on Xeon).

Procs	Cores	Freq	Time [s]	Energy [Wh]	Energy [J]	AvgPower [W]
1	1	2.20GHz	384.14	8.46	30,456	79.48
1	2	2.20GHz	195.63	4.60	16,560	84.93
1	3	2.20GHz	138.33	3.36	12,096	88.32
1	4	2.20GHz	106.39	2.71	9756	92.10
1	5	2.20GHz	89.29	2.31	8316	94.73
1	6	2.20GHz	77.76	2.08	7488	97.18
1	7	2.20GHz	69.07	1.87	6732	99.04
1	8	2.20GHz	62.09	1.73	6228	100.73
1	9	2.20GHz	55.23	1.58	5688	103.89
1	10	2.20GHz	49.65	1.46	5256	107.49
1	11	2.20GHz	49.64	1.46	5256	107.85
1	12	2.20GHz	45.94	1.37	4932	109.42
1	13	2.20GHz	42.85	1.28	4608	110.22
1	14	2.20GHz	40.12	1.20	4320	110.81
1	15	2.20GHz	37.48	1.14	4104	112.08
1	16	2.20GHz	35.42	1.10	3960	112.64
1	17	2.20GHz	34.02	1.06	3816	112.98
1	18	2.20GHz	31.58	0.97	3492	113.88
1	19	2.20GHz	29.93	0.92	3312	114.54
1	20	2.20GHz	28.83	0.89	3204	115.21

For heterogeneous systems, such as XU3 and TX2, we provide four measured data sets per application, as exemplified in Table 4, Table 5, Table 6, Table 7 for EP on XU3. The first two data sets represent the execution with OpenMP static scheduling on big and little cores, respectively. The last two data sets represent the execution on all cores using static and dynamic OpenMP execution, respectively.

**Table 4:** Raw time-energy measurements on big cores with static scheduling (EP on XU3).

#Procs	Cores	Freq	Time [s]	Energy [Wh]	Energy [J]	AvgPower [W]
1	1	2.00GHz	710.81	0.02	60.12	9.27
1	2	2.00GHz	363.58	0.01	42.12	12.51
1	3	2.00GHz	250.09	0.01	36.00	15.03
1	4	2.00GHz	197.88	0.01	29.52	15.56

**Table 5:** Raw time-energy measurements on little cores with static scheduling (EP on XU3).

#Procs	Cores	Freq	Time [s]	Energy [Wh]	Energy [J]	AvgPower [W]
1	1	2.00GHz	1607.79	0.03	99.00	6.65
1	2	2.00GHz	820.67	0.01	53.28	7.10
1	3	2.00GHz	548.60	0.01	37.80	7.51
1	4	2.00GHz	413.19	0.01	29.88	7.89

**Table 6:** Raw time-energy measurements on all cores with static scheduling (EP on XU3).

#Procs	Cores	Freq	Time [s]	Energy [Wh]	Energy [J]	AvgPower [W]
1	1	2.00GHz	714.61	0.017	61.2	9.24
1	2	2.00GHz	363.75	0.012	43.2	12.45
1	3	2.00GHz	250.87	0.01	36	14.69
1	4	2.00GHz	198.74	0.008	28.8	15.32
1	5	2.00GHz	321.7	0.01	36	10.94
1	6	2.00GHz	273.5	0.008	28.8	11.10
1	7	2.00GHz	235.14	0.007	25.2	11.35
1	8	2.00GHz	206.75	0.007	25.2	11.67

**Table 7:** Raw time-energy measurements on all cores with dynamic scheduling (EP on XU3).

#Procs	Cores	Freq	Time [s]	Energy [Wh]	Energy [J]	AvgPower [W]
1	1	2.00GHz	709.82	0.017	61.2	9.25
1	2	2.00GHz	364.94	0.012	43.2	12.47
1	3	2.00GHz	248.67	0.01	36	15.11
1	4	2.00GHz	198.96	0.008	28.8	15.31
1	5	2.00GHz	179.27	0.007	25.2	15.32
1	6	2.00GHz	162.8	0.007	25.2	15.41
1	7	2.00GHz	149.53	0.006	21.6	15.54
1	8	2.00GHz	137.73	0.006	21.6	15.67

### 2.3 Model output data

Our analytic models [1,2] are implemented in Python and can be run on a Linux system using the provided bash scripts. There are two wrapper scripts corresponding to homogeneous and heterogeneous systems, respectively. Besides speedup and energy data, these scripts take as parameters the number of cores, the active power fraction (APF) [1,2] and the idle power of the system. By tweaking these parameters, users can explore new system designs and estimate their time-energy efficiency.

Model output data consists of nine columns, as shown in Table 8 for EP running on Xeon when Amdahl's law [3] for speedup is used. The first column represents the number of cores used for execution, while the other eight columns represent measured and predicted speedup, energy savings, execution time and energy, respectively.

**Table 8:** Model output data (EP on Xeon, Amdahl's law).

Cores	Measured Speedup	Predicted Speedup	Measured Energy Savings	Predicted Energy Savings	Measured Time	Predicted Time	Measured Energy	Predicted Energy
1	1	1	0	0	384.1	384.1	30,530	24,233.8
2	1.96	1.94	0.456	0.464	195.6	198	16,615.1	13,024.5
3	2.78	2.82	0.6	0.619	138.3	136	12,217.2	9288
4	3.61	3.66	0.679	0.696	106.4	105	9798.4	7419.8
5	4.3	4.45	0.723	0.743	89.3	86.4	8458.3	6298.9
6	4.94	5.19	0.752	0.773	77.8	73.9	7556.8	5551.6
7	5.56	5.9	0.776	0.796	69.1	65.1	6841	5017.8
8	6.19	6.57	0.795	0.812	62.1	58.4	6254.2	4617.5
9	6.96	7.21	0.812	0.825	55.2	53.3	5737.6	4306.1
10	7.74	7.82	0.825	0.835	49.6	49.1	5336.9	4057
11	7.74	8.4	0.825	0.844	49.6	45.7	5353.9	3853.2
12	8.36	8.95	0.835	0.851	45.9	42.9	5026.8	3683.4
13	8.96	9.47	0.845	0.857	42.9	40.5	4723	3539.7
14	9.57	9.98	0.854	0.862	40.1	38.5	4445.6	3416.5
15	10.25	10.46	0.862	0.866	37.5	36.7	4200.6	3309.7
16	10.85	10.92	0.869	0.87	35.4	35.2	3989.6	3216.3
17	11.29	11.36	0.874	0.874	34	33.8	3843.4	3133.9
18	12.16	11.79	0.882	0.877	31.6	32.6	3596.3	3060.6
19	12.83	12.19	0.888	0.879	29.9	31.5	3428.1	2995.1
20	13.32	12.59	0.891	0.882	28.8	30.5	3321.6	2936.1

In addition, the source code implementing the model reports the sequential fraction and the Root-Mean-Square Deviation (RMSD) between measured and predicted values across all core counts. A summary consisting of the sequential fraction (f), RMSD of the sequential fraction (RMSD(f)) and RMSD of energy savings (RMSD (es)) for each workload and for both Amdahl's and Gustafson's laws, is written in a *stats.csv* file for each system. Table 9 exemplifies such data for the Xeon system.

**Table 9:** Model accuracy output (on Xeon).

#Val	f		RMSD(f)		RMSD (es)	
#App	Amdahl	Gustafson	Amdahl	Gustafson	Amdahl	Gustafson
EP	0.03	0.33	0.373	0.406	1.3	2.3
LV	0.05	0.42	0.26	0.664	1.4	3.3
BT	0.1	0.62	0.512	1.09	3.1	7.5
SP	0.22	0.81	0.583	1.142	10.3	16.6
BS	0.06	0.5	0.249	0.594	1.2	4.5
KM	0.38	0.89	0.168	0.306	9.5	11.3
PF	0.46	0.93	0.036	0.305	2.2	11.8
CL	0.2	0.79	0.456	1.011	8.5	13.9
FE	0.19	0.78	0.284	0.902	7.4	11.5
GH	0.99	0.9999	0.028	0.029	2.6	2.5

**Fig. 2:**
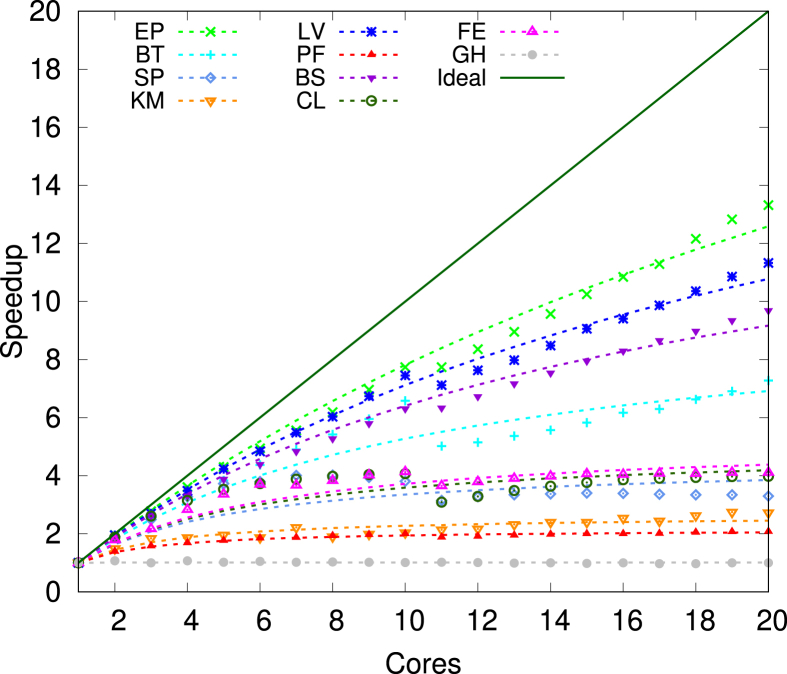
Amdahl speedup on Xeon.

**Fig. 3:**
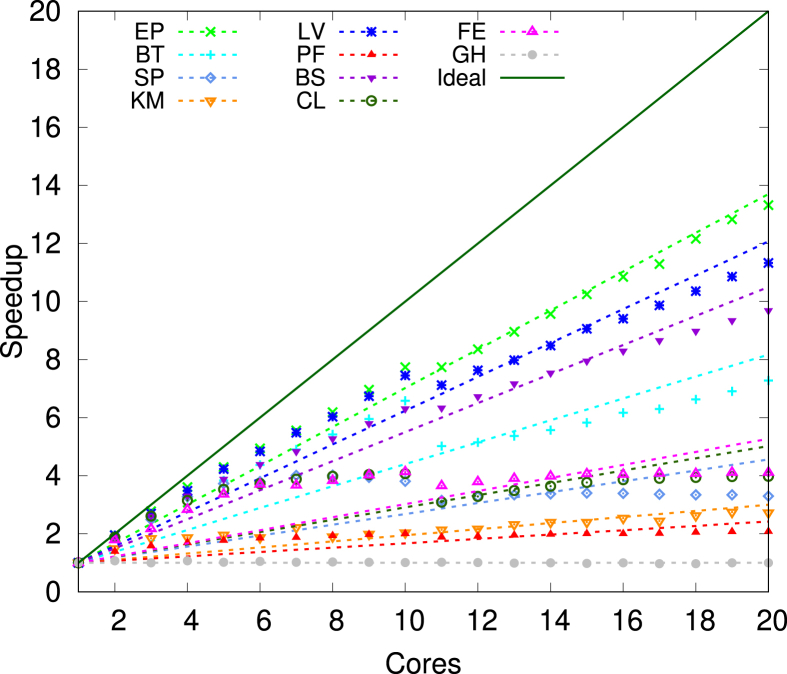
Gustafson speedup on Xeon.

The speedup values in Table 8 correspond to Amdahl's law [3] and are used to plot Fig. 2. On the other hand, Fig. 3 represents the same measurements, while the predicted speedup is determined using Gustafson's law [4]. The results for other systems are presented in our research papers [1,2].
